# Closure Devices for Iatrogenic Thoraco-Cervical Vascular Injuries

**DOI:** 10.1007/s00270-016-1506-z

**Published:** 2016-11-28

**Authors:** Gregory C. Makris, Rafiuddin Patel, Mark Little, Carina Tyrrell, James Sutcliffe, Kader Allouni, Mark Bratby, Susan Anthony, Raman Uberoi

**Affiliations:** 1 Interventional Radiology Department, Oxford University Hospitals, NHS Foundation Trust, Oxford, UK; 2 Alfa Institute of Biomedical Sciences, Neapoleos 9, Athens, Greece

**Keywords:** Closure device, Iatrogenic, Vascular injury, Interventional radiology

## Abstract

**Introduction:**

The unintentional arterial placement of a central venous line can have catastrophic complications. The purpose of this systematic review is to assess and analyse the available evidence regarding the use of the various vascular closure devices (VCDs) for the management of iatrogenic thoraco-cervical arterial injuries (ITCAI).

**Methods:**

A systematic review was performed according to PRISMA guidelines.

**Results:**

Thirty-two relevant case series and case reports were identified with a total of 69 patients having being studied. In the majority of the studies, plug-based VCDs were used (81%) followed by suture-based devices (19%). The majority of studies reported successful outcomes from the use of VCDs in terms of achieving immediate haemostasis without any acute complications. Long-term follow-up data were only available in nine studies with only one case of carotid pseudoaneurysm being reported after 1-month post-procedure. All other cases had no reported long-term complications. Five studies performed direct or indirect comparisons between VCDs and other treatments (open surgery or stent grafting) suggesting no significant differences in safety or effectiveness.

**Conclusion:**

Although there is limited evidence, VCDs appear to be safe and effective for the management of ITCAIs. Further research is warranted regarding the effectiveness of this approach in comparison to surgery and in order to identify those patients who are more likely to benefit from this minimally invasive approach.

## Introduction

**Fig. 1:**
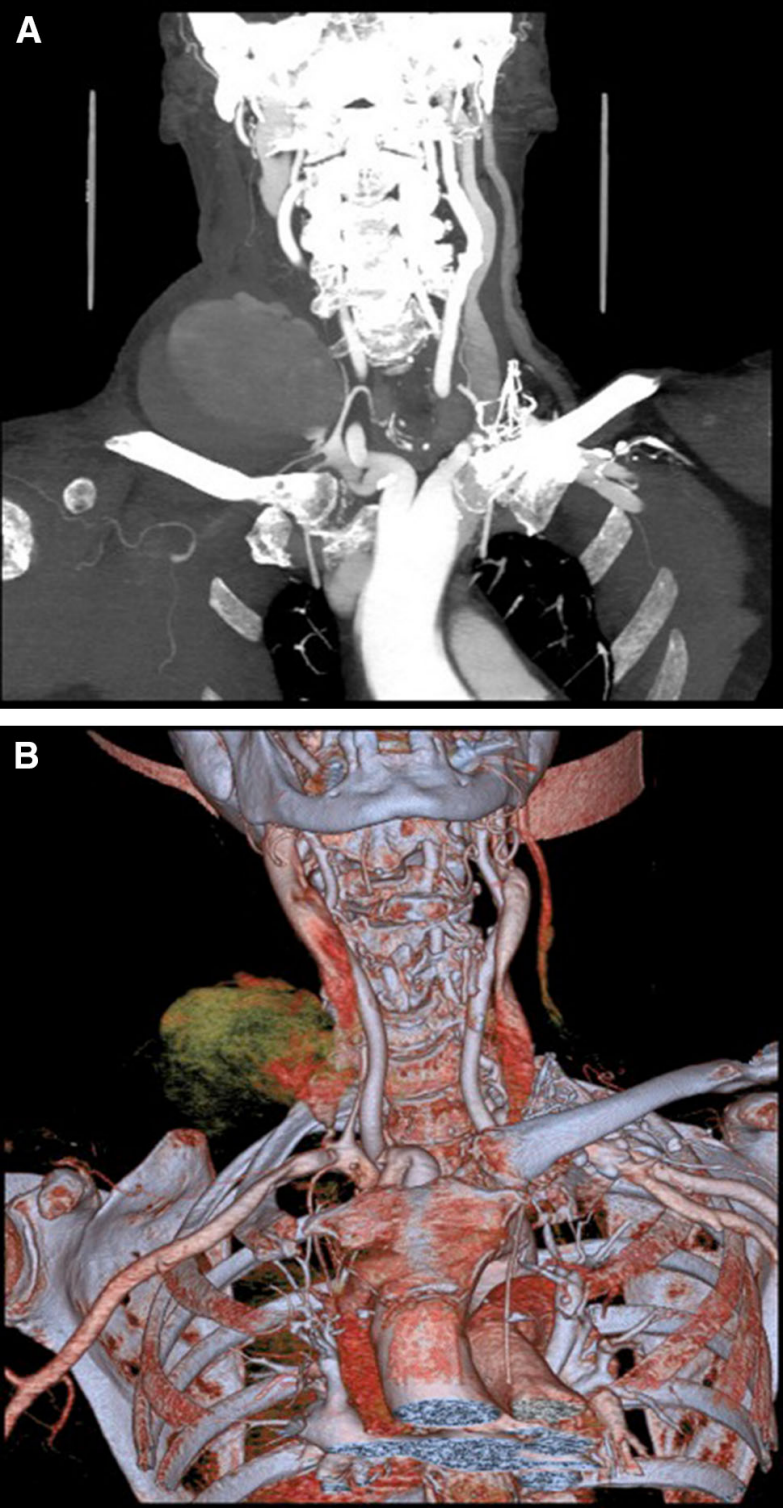
Coronal CT (**A**) and 2D reconstruction (**B**) of the upper chest and neck demonstrating large haematoma after inadvertent right subclavian artery puncture during right internal jugular vein line placement

Central venous line insertion is a common medical procedure that can be complicated by inadvertent arterial placement of the catheter. This can be the cause of significant mortality and morbidity for the patient [1]. Treatment options include open surgical repair, compression, off-label use of percutaneous vascular closure devices (VCDs), and/or stent grafts. Some of the potential complications include bleeding, thrombosis, stroke, limb ischaemia, neurologic deficit, and death (Fig. 1). [1].

The use of VCDs in interventional radiology has revolutionised the way we achieve haemostasis offering a safe and effective alternative to manual compression. At the same time they have made endovascular abdominal aneurysm repair a truly minimally invasive (percutaneous) procedure very often without the need for time-consuming groin cut-downs. There are many different types of VCD that offer solutions for a variety of indications and vascular disease profiles. The main types of VCDs include suture based, plug based, and nitinol clips [2]. These sophisticated devices have been shown to have a good safety profile for closure of arteriotomies post endovascular procedures with overall rates of complications similar between manual compression at 13.1% and VCDs at 12.2% [3].

The off-label use of these interventional radiology devices for the treatment of iatrogenic injuries of thoraco-cervical vascular injuries (ITCVI) post central venous line placement is becoming increasingly common. The location and the local anatomy where these vascular injuries occur (carotid, brachiocephalic, subclavian, or vertebral arteries) make the use of manual compression difficult or even dangerous [3, 4]. Traditionally these cases have been treated with an open surgical repair and more recently with the placement of a stent graft where possible [4].

The purpose of this study is to review the available clinical evidence regarding the safety and effectiveness of the available VCDs for the management of ITCVI.

## Methods

A systematic review was performed according to PRISMA guidelines [5]. The PubMed, Scopus, and Cochrane databases were searched for clinical studies evaluating the short- and long-term clinical outcomes from the use of VCDs for the treatment of iatrogenic thoraco-cervical vascular injuries. The search terms used were: “closure device”, “interventional radiology”, AND “subclavian” OR “carotid” OR “vertebral” OR “iatrogenic” OR “thoraco-cervical” in various combinations. Two independent reviewers GCM and ML performed the literature search and data extraction.

The selected studies were manually searched for relevant publications out of their reference lists. All clinical studies, which reported results on safety and effectiveness of VCDs for the treatment of iatrogenic vascular injuries were retrieved and analysed. In vitro or animal-only studies were excluded from the analysis. Due to the small number of relevant studies, case report studies were also included. There was no language or time limit to our search. The end date of this search was 29th of August 2016.

## Results

### Systematic Review of the Available Evidence

**Fig. 2:**
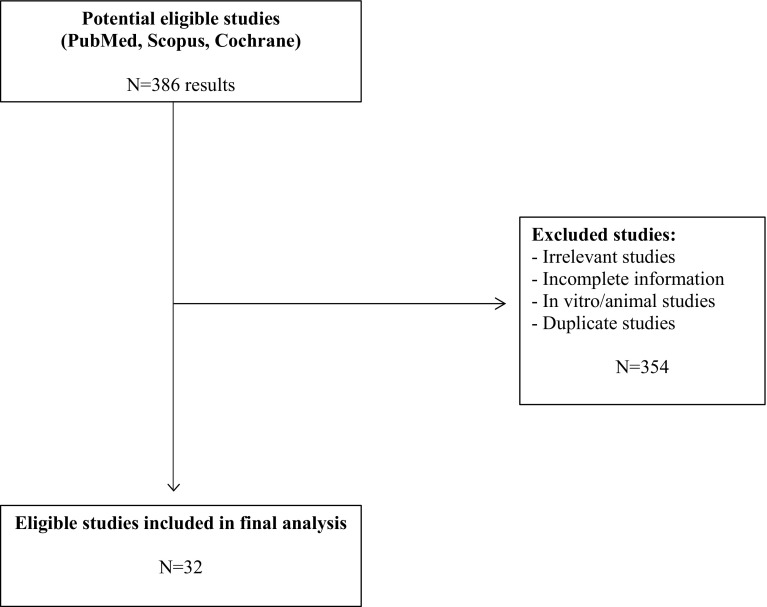
Flowchart describing the selection of eligible studies for this systematic review

The database search returned 382 results out of which 32 studies [3, 6–35] were eligible for inclusion in this review (Fig. 2) with a total of 69 patients having being studied (Table 1). All studies were retrospective case series or case reports with small sample sizes. There were six case series studies (5–8 patients) with the remaining studies being cases reports (1–3 patients). Iatrogenic injury in carotid arteries was evaluated in ten studies, 25 studies assessed subclavian injuries whereas brachiocephalic injuries were assessed in one study (Table 1). The size of the catheters causing the iatrogenic injury ranged between 6 and 12F, with the majority being between 6 and 9F.

**Table 1:** Clinical studies reporting the use of closure devices for the treatment of iatrogenic thoraco-cervical vascular injuries

Author (year) [Ref]	Study design	No patients	Target vessel (size of catheter)	Type of closure device	Outcome (complications)	Follow-up (outcome)		Compared to surgery
Cuellar (2015) [6]	Retrospective study	8	CA (6 or 8)	Angioseal	Good haemostasis (no complications)		6 m (no complications)	ND
Bechara (2013) [7]	Retrospective study	6 (& 6 had surgical repair)	CA (8.5)	Suture-mediated closure device	Good haemostasis (no complications)		42 m (no complications)	CDs were safe and effective as open repair
Powers (2011) [8]	Retrospective study	1 (out of 13 studied pts)	CA (7)	Angioseal	Good haemostasis (no complications)		ND	ND
Stellmes (2014) [9]	Case series	5	CA and SA (7, 8.5, 9)	Starclose or exoseal	Good haemostasis (no complications)		1 m (pseudoaneurysm)	ND
Schütz (2011) [10]	Case report	1	CA (8)	Angioseal	Good haemostasis (no complications)		ND	ND
Massière (2009) [11]	Case report	1	CA (8)	Angioseal	Good haemostasis (no complications)		ND	ND
Marina (2007) [12]	Case report	1	CA (8)	Angioseal	Good haemostasis (no complications)		ND	ND
Blanc (2002) [13]	Case report	1	CA (6)	Angioseal	Good haemostasis (no complications)		US and angio^a^ (no complications)	ND
Kirkwood (2008) [14]	Case report	3	CA (7)	Angioseal	Good haemostasis (no complications)		ND	ND
Guilbert (2008) [15]	Retrospectively study	2 (out of 13 pts)	CA or SA (≥7)	Angioseal	Good haemostasis (no complications)		ND	CDs were safe and effective as open repair
Tran (2009) [16]	Retrospective study	5	SA	Starclose	Good haemostasis (no complications)		ND	ND
Shetty (2007) [17]	Case report	1	SA (8)	Angioseal	Good haemostasis (no complications)		ND	ND
Fraizer (2003) [18]	Case report	1	SA (8.5)	Suture-based device	Good haemostasis (no complications)		ND	ND
Sharma (2008) [19]	Case report	1	SA (7)	Collagen-based vascular device	Occlusion of the artery		ND	ND
Redmond (2014) [20]	Case report	1	SA	Angioseal	Good haemostasis (no complications)		ND	ND
Cohen (2014) [21]	Case report	1	SA (4)	Angioseal	Good haemostasis (no complications)		ND	ND
Mousa (2015) [22]	Case report	1 (out of 3 pts)	SA	Angioseal	Good haemostasis (no complications)		ND	ND
Szkup (2012) [23]	Case report	2	SA (7)	Angioseal	Good haemostasis (no complications)		ND	ND
Devriendt (2009) [24]	Case report	1	SA (7.5)	Angioseal	Good haemostasis (no complications)		ND	ND
Guimaraes (2008) [25]	Case series	5	SA and BCA (7)	Angioseal	Good haemostasis (no complications)		12.5 m (no complications)	ND
Micha (2007) [26]	Case report	1	SA (6)	Angioseal	Good haemostasis (no complications)		ND	ND
Wildberger (2006) [27]	Case report	1	SA (12)	VasoSeal^b^	Good haemostasis (no complications)		ND	ND
Wallace (2001) [28]	Case report	1	SA	Prostar XL^b^	Good haemostasis (no complications)		ND	ND
Berlet (2001) [29]	Case report	2	SA (6 and 12)	Proglide	Good haemostasis (no complications)		ND	ND
OUH (2015)^c^	Individual cases	3	CA (7)	Proglide	Good haemostasis (no complications)		6–24 m (no complications)	ND
Yoon (2015) [3]	Case series	4 (out of 13 pts)	CA, SA (7 or 8)	Angioseal	Good haemostasis (overall complication rate was 7%^d^)		36 months (no complications)	CDs were safe and effective as operative repair.
Micha (2006) [30]	Case report	1	SA (6)	Angioseal	Good haemostasis (no complications)		ND	ND
Nicholson (2004) [31]	Retrospective study	4 (out of 9 pts)	SA (7 or 10)	Angioseal	Good haemostasis (no complications)		1 to 12 months (no complications)	ND
Railo (2004) [32]	Case report	1	SA (11.5)	Angioseal	Good haemostasis (no complications)		3 months (no complications)	ND
Molnár (2008) [33]	Case report	1	SA (9)	Angioseal	Good haemostasis (no complications)		9 months (no complications)	ND
Bovenschul (2007) [34]	Case report	1	SA (7)	Angioseal	Good haemostasis (no complications)		ND	ND
Bovenschulte (2011) [35]	Case report	1	SA (13)	Angioseal	Good haemostasis (no complications)		ND	ND

*CA* carotid artery, *SA* subclavian artery, *BCA* brachiocephalic artery, *ND* no data, *OUH* Oxford university hospital experience, *f/u* follow-up, *pts* patients, *m* months, *US* ultrasound, *angio* angiogram, *No* number of

^a^Unspecified time of follow-up

^b^Temporary balloon tamponade was also performed

^c^Unpublished data by the Interventional Radiology Department of Oxford University Hospital, UK

^d^1 out of 13

A number of VCDs were used with the majority being AngioSeal (St. Jude Medical, MN, USA), (12 studies). Other collagen-based VCDs [Vasoseal (Datascope, NJ, USA)] and Exoseal (Cordis Corp., NJ, USA) were used in three studies with a total of four patients. Suture-based VCDs were trialled in five studies (12 patients with Proglide and one with Prostar (Abbott Vascular, IL, USA)). Nitinol clips were used in one study (Starclose, Abbott Vascular, IL, USA) and a total of seven patients. Finally, in two studies [27, 28] the VCDs were used in conjunction with temporary balloon tamponade. All included studies reported safety and effectiveness outcomes for the acute phase post-procedure with only nine studies [3, 6, 7, 9, 13, 25] reporting long-term follow-up data (1–42 months).

The majority of studies reported good outcomes from the use of VCDs in terms of achieving immediate haemostasis without any acute complications (98%). There was only one case report [19] where there was an acute total subclavian artery occlusion as a result of the use of a collagen-based vascular closure device. In this case, prompt angiography and balloon inflation via an already present sheath in the brachiocephalic artery resulted in the restoration of flow. All other cases (68 patients) had no reported acute complications. Long-term follow-up data were only available in nine studies [3, 6, 7, 9, 13, 25, 31–33] with only one case of carotid pseudoaneurysm being reported after 1-month post-procedure. All other cases (31 patients-97%) had no long-term complications reported. However, the follow-up time varied significantly, ranging from 1 to 42 months.

Three studies were identified having performed direct or indirect comparisons between VCDs versus other treatments [3, 7, 15]. One study [3] directly compared VCDs with open surgery concluding that both were equally safe and effective, though VCDs offered benefits in terms of treatment duration (6 vs. 32 min *p* = 0.03) and mean delays to operation (3 vs. 5 days, *p* = 0.20). In two other studies, patients were treated with VCDs, open surgery or stent grafting or embolization of the injured vessel [7, 15]. Good haemostasis and no acute complications were mentioned with the above techniques, however there were no data regarding long-term outcomes and cost-effectiveness.

## Discussion

The purpose of this systematic review was to review and assess the available evidence around the use of VCDs for the management of ICTV injuries. Currently VCDs are widely used in interventional radiology to reduce complications from arterial access and reduce cost.

A recent meta-analysis included a total of 34 studies and 14,401 patients, where 5659 patients underwent manual compression and 8742 patients underwent vascular closure device placement. Overall, the rate of procedural success for VCD patients was 95.7%. The overall median time to haemostasis for manual compression was 22.9 min compared to VCDs at 5.95 min. The study found that 94.4% of patients randomized to the vascular closure device group who had undergone prior angioplasty preferred the use of VCDs if a further angioplasty was to be performed in the future [36].

The off-label use of VCDs for the treatment of iatrogenic vascular injuries is not as well studied. However, there is an increasing number of papers suggesting that VCDs can be a viable alternative to open surgery for iatrogenic vessel injury. A previous study by Blair et al. [4] showed that the incidence of complications was highly different between pull/pressure technique vs. the surgical or endovascular approach, with a relative risk of 17.8 favouring surgical or endovascular repair (*P* < .001) and a number needed to treat of 1.5 (1.3–2.4).

Indeed, the presented data on this review suggest that VCDs used by an experienced operator can be a safe treatment modality for iatrogenic thoraco-cervical injuries with comparable results to open surgery, which currently appears to be the preferred choice of treatment. Both statements are only supported by case reports and case series and especially the latter statement was only assessed in three small studies (total of 12 patients). In addition, there was great variation in terms of the type of VCDs used and it was not possible to perform comparisons between them. The majority of the VCDs used in the studies included in this review were plug based followed by suture based.

Our institutional experience is similar to the majority of the reported outcomes as they were previously presented. Within the last 2 years we had three cases of ITCVI when the interventional radiology department was asked to assess and intervene. All three cases were successfully treated with a proglide closure device over a J-wire and with no immediate complications. The long-term follow-up of these cases (minimum 6 months, maximum 2 years follow-up) was performed with ultrasound evaluation and was unremarkable.

There are a number of significant limitations in this systematic review. Most studies are case reports or case series with a very small sample size and with considerable heterogeneity. Only a small number of studies performed comparisons between surgical and interventional radiology treatments and even in those studies the number of patients were too few to reach any solid conclusions. Comparison between the types of injured vessel was not possible due to the limited number of studies. In addition it is possible that publication bias has influenced the number of available case reports or case series that could have potentially contributed more negative data on the use of VCDs. Other types of catheter causing injury were not included, for example, dialysis catheters.

It has to be noted that no definitive guidelines were identified from our search with regards to the management of these simple but potentially catastrophic iatrogenic injuries. A large variation in the management plan between various institutions was noted without clear indications when open surgery or endovascular treatments is preferred. Some authors believe that endovascular treatments (covered stent grants, percutaneous VCDs) may offer good results when selected appropriately based on imaging evaluation, whereas for more complex cases with associated pseudoaneurysms and/or AVFs an open repair may be necessary [3]. However, in this review we presented two case reports where relatively large AVFs [13, 25] were treated with VCDs.

In light of the above evidence we believe that the management of these potentially life-threatening complications should become more formalised with more emphasis given on the need to increase awareness among the involved medical stuff. The use of VCDs for the management of this type of complication is supported by the current literature, however the number of the studied cases is small in order to make a solid case. Until more evidence is available, the management of iatrogenic injuries of thoraco-cervical vascular injuries should be jointly performed by both interventional radiologists and vascular surgeons, in order to carefully assess the risks of every individual case. More research is warranted to assess the long-term effectiveness of this approach as well as its safety when compared to open surgical repair.

## Conclusion

VCDs in the treatment of iatrogenic injury of the major thoraco-cervical arteries can be a very attractive option. Although the number of treated patients is small, this technique appears to be safe and effective. Interventional radiologists and vascular surgeons should work closely to decide the optimal management of these patients. Careful evaluation of the post-injury vascular imaging and consideration of the relative merits of minimally invasive, open surgical or non-invasive treatment for the individual patient on a case-by-case basis should always be performed.
